# Erratum: Triplet *p*-wave pairing correlation in low-doped zigzag graphene nanoribbons

**DOI:** 10.1038/srep46879

**Published:** 2017-08-29

**Authors:** Tianxing Ma, Fan Yang, Zhongbing Huang, Hai-Qing Lin

Scientific Reports 7: Article number: 42262; 10.1038/srep42262 published online: 02 10 2017; updated: 08 29 2017.

This article was published twice in error during a change in production systems. The publisher apologizes to the authors and readers for the error. When citing this work, please refer to the original version.^1^
